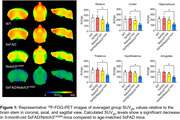# Cerebrovascular and Metabolic Alterations in a Mouse Model of Familial Alzheimer’s Disease Carrying the Notch3 C456R Mutation

**DOI:** 10.1002/alz.091419

**Published:** 2025-01-09

**Authors:** Leila Letica, Sang‐Ho Choi, Lauren R Dubberley, Diego Szczupak, Jung Eun Park, David J Schaeffer, Afonso C Silva

**Affiliations:** ^1^ University of Pittsburgh School of Medicine, Pittsburgh, PA USA

## Abstract

**Background:**

Vascular pathology associated with small vessel disease (SVD), such as microinfarcts and microbleeds, are common in elderly populations and significant contributors to cognitive impairment and dementia. Autosomal dominant cerebral arteriopathy with subcortical infarctions and leukoencephalopathy (CADASIL), caused by mutations in the Notch3 gene, is the most prominent inheritable SVD, with a common etiology of subcortical strokes and dementia. This study aimed to investigate additive or synergistic effects of CADASIL‐related vascular alterations and familial Alzheimer’s disease (FAD)‐related amyloid pathology on cerebral metabolism of glucose and disease progression in a novel FAD‐CADASIL mouse model.

**Methods:**

We bred 5xFAD mice to CADASIL mice carrying the Notch3 C456R mutation to create the novel FAD‐CADASIL mouse model. To investigate progressive alterations in cerebral glucose metabolism, ^18^F‐FDG was delivered to awake mice via a tail vein injection, with an average dose of 18.5 MBq. The mice were fasted for a minimum of 2 hours before ^18^F‐FDG administration. PET and CT acquisitions were completed after a 30‐minute ^18^F‐FDG circulation period using a Bruker Si78 instrument. Static datasets were analyzed for standard uptake values relative to the brainstem (SUV_r_) and corrected for glucose levels (SUV_glc_). Statistical tests included unpaired t‐tests and 1‐ & 2‐way ANOVA using GraphPad Prism 9.

**Results:**

Whole brain SUV_r_ values were decreased across age groups in 5xFAD, Notch3^C456R^, and 5xFAD/Notch3^C456R^ mice compared to age‐matched WT controls. Blood glucose levels collected before ligand injection indicated an age‐dependent decrease in glucose levels across all genotypes. 5xFAD/Notch3^C456R^ mice showed distinct reductions in whole brain SUV_glc_ values compared to aged‐matched 5xFAD and Notch3^C456R^ mice. ROI analysis of 3‐month‐old 5xFAD/Notch3^C456R^ shows significantly decreased SUV_glc_ levels in several cortical regions such as the striatum, cortex, hippocampus, hypothalamus, and thalamus when individually compared to WT, 5xFAD, and Notch3^C456R^ mice (Figure 1).

**Conclusion:**

We observed a significant synergistic effect of CADASIL on accelerating increased amyloid accumulation and reducing cerebral glucose metabolism in the novel FAD‐CADASIL mouse model. These results provide further key evidence of an association between cortical vascular pathology and Alzheimer’s Disease progression.

This research was funded by NIH/NINDS grant RF1NS117486.